# Metabolic classification of circulating tumor cells as a biomarker for metastasis and prognosis in breast cancer

**DOI:** 10.1186/s12967-020-02237-8

**Published:** 2020-02-06

**Authors:** Jing Chen, Changsheng Ye, Jianyu Dong, Shunwang Cao, Yanwei Hu, Bo Situ, Xiaoxue Xi, Sihua Qin, Jiasen Xu, Zhen Cai, Lei Zheng, Qian Wang

**Affiliations:** 1 Department of Laboratory Medicine, Zhujiang Hospital, Southern Medical University, Guangzhou, 510515 China; 2 Laboratory Medicine Center, Nanfang Hospital, Southern Medical University, Guangzhou, 510000 China; 3 Department of Breast Surgery, Nanfang Hospital, Southern Medical University, Guangzhou, 510000 China; 4 Department of Laboratory Medicine, The Second Affiliated Hospital of Guangzhou University of Chinese Medicine, Guangzhou, 510000 China; 5 Clinical Laboratory, Guangzhou Women and Children’s Medical Center, Guangzhou Medical University, Guangzhou, 510000 China; 6 SurExam Bio-Tech, Guangzhou Technology Innovation Base, Science City, Guangzhou, 510000 China

**Keywords:** Circulating tumor cells typing, Metabolic reprogramming, Breast cancer

## Abstract

**Background:**

Circulating tumor cells (CTCs) has been demonstrated as a promising liquid biopsy marker for breast cancer (BC). However, the intra-patient heterogeneity of CTCs remains a challenge to clinical application. We aim at profiling aggressive CTCs subpopulation in BC utilizing the distinctive metabolic reprogramming which is a hallmark of metastatic tumor cells.

**Methods:**

Oncomine, TCGA and Kaplan–Meier plotter databases were utilized to analyze expression and survival relevance of the previously screened metastasis-promoting metabolic markers (PGK1/G6PD) in BC patients. CTCs detection and metabolic classification were performed through micro-filtration and multiple RNA in situ hybridization using CD45 and PGK1/G6PD probes. Blood samples were collected from 64 BC patients before treatment for CTCs analysis. Patient characteristics were recorded to evaluate clinical applications of CTCs metabolic subtypes, as well as morphological EMT subtypes classified by epithelial (EpCAM/CKs) and mesenchymal (Vimentin/Twist) markers.

**Results:**

PGK1 and G6PD expressions were up-regulated in invasive BC tissues compared with normal mammary tissues. Increased tissue expressions of PGK1 or G6PD indicated shortened overall and relapse-free survival of BC patients (*P* < 0.001). Blood GM^+^CTCs (DAPI^+^CD45^−^PGK1/G6PD^+^) was detectable (range 0–54 cells/5 mL) in 61.8% of tCTCs > 0 patients. Increased GM^+^CTCs number and positive rate were correlated with tumor metastasis and progression (*P* < 0.05). The GM^+^CTCs ≥ 2/5 mL level presented superior AUC of ROC at 0.854 (95% CI 0.741–0.968) in the diagnosis of BC metastasis (sensitivity/specificity: 66.7%/91.3%), compared with that of tCTCs (0.779) and CTCs-EMT subtypes (E-CTCs 0.645, H-CTCs 0.727 and M-CTCs 0.697). Moreover, GM^+^CTCs^+^ group had inferior survival with decreased 2 years-PFS proportion (18.5%) than GM^+^CTCs^−^ group (87.9%; *P* = 0.001).

**Conclusions:**

This work establishes a PGK1/G6PD-based method for CTCs metabolic classification to identify the aggressive CTCs subpopulation. Metabolically active GM^+^CTCs subtype is suggested a favorable biomarker of distant metastasis and prognosis in BC patients.

## Background

Breast cancer (BC) is the most common cancer in women and accounts for 24.2% of female new cases worldwide (Global Cancer Statistics 2018) [1]. Although the development of modern medical technology has improved the therapeutic effect of BC, tumor-related death caused by metastasis and recurrence remains major trouble in clinical management. Recently, circulating tumor cells (CTCs) attracts widespread concern as a biomarker for prognosis and monitoring in BC [2–4] and other tumors [5, 6], benefiting from the easy operation of sampling compared with tissue biopsy. However, recent experimental studies presented that only a few CTCs population could succeed in metastases formation in mouse models, revealing intra-patient heterogeneity of CTCs [7, 8]. How to identify the aggressive CTCs subpopulation is an urgent yet challenging problem. Phenotypic analysis of CTCs could throw light on their outcome in metastasis to further provide information for disease assessment.

Research on molecular phenotypes of CTCs has emerged over the last decade. The occurrence of epithelial-mesenchymal transition (EMT) in CTCs [9, 10] results in epithelial-, mesenchymal- and hybrid-subtypes of CTCs. The clinical analysis showed that mesenchymal- and hybrid-CTCs predicted higher metastasis risk and shorter relapse-free survival (RFS) than epithelial-CTCs [11, 12], demonstrating that CTCs undergoing EMT are behaviorally more invasive. Variations in the expression of hormone receptors (ER/PR), HER-2 and CA15-3 in tumor samples are also investigated as classification markers of CTCs [13–15]. Aktas et al. [13] profiled ER/PR/HER-2 expression in metastatic BC patients and observed 43–67% of the concordant expression of these markers between CTCs and metastases. Nonetheless, controversy exists about the disease relevance of these CTCs subtypes such as EMT classification, due to the dynamic EMT balance involved in complex metastasis cascade [16]. The mesenchymal features of tumor cells might disappear once new colonization occurs. Hence, it is desirable to develop new markers to profile the activity and function of CTCs.

Metabolic reprogramming is a vital feature of cancer cells, characterized by enhanced glycolysis, the pentose phosphate pathway, and glutaminolysis [17]. The reprogrammed metabolism equipped tumor cells with the rapid generation of energy and precursor molecules, which are essential for cell proliferation and metastasis. Our early research and other’s report demonstrated that the metabolic transition from oxidative phosphorylation to aerobic glycolysis plays a vital role in cell invasion [18, 19]. Accordingly, we hypothesize that the metabolic features could be functional markers for CTCs classification, considering the decisive roles of the metabolic switch in cell behavior. Previously we screened the differentially expressed metabolic genes in high and low metastatic cancer cells. PGK1 and G6PD were found closely correlated with the metastatic potential of tumor cells, and CTCs from prostate cancer patients presented heterogeneous expression of these genes [20]. Increased expression of PGK1 and G6PD, as critical regulators involved in glycolysis and the pentose phosphate pathway, respectively, reveals active glucose metabolism of tumor cells. Studies demonstrated that up-regulated PGK1 and G6PD in BC tissues is associated with a high risk of recurrent metastasis and poor survival of patients. The mechanisms include the enhancement of energy supply and activation of the HIF-1α pathway in cell migration and invasion [21, 22]. Therefore, PGK1 and G6PD are potential markers to profile the metabolic activity of CTCs and further indicate the functional CTCs subtypes for BC patients. The clinical relevance of these subtypes remains to be explored.

In this work, we aim to establish a metabolic typing method for CTCs based on the combined PGK1/G6PD markers and investigate the clinical significance of CTCs metabolic classification in BC. Characterization of CTCs metabolism is expected to bring to light the metabolic and functional heterogeneities of CTCs, and further promote the better application of CTCs phenotypic analysis.

## Materials and methods

### Oncomine and TCGA database expression analysis

The mRNA level of PGK1 and G6PD in BC tissue was analyzed using the Oncomine (http://www.oncomine.org) [23] and The Cancer Genome Atlas (TCGA) (http://www.cancer.gov/tcga) [24] database. Oncomine is a microarray database consisted of gene data from 715 datasets and 86,733 samples. We set search filters as *Cancer vs. Normal Analysis* (analysis type), *Breast Cancer* (cancer type), and *P *<* 0.05*. Among the candidate datasets, we chose Curtis Breast which possessed the largest sample size (http://www.ebi.ac.uk/ega/studies/EGAS00000000083). In 1556 invasive ductal BC tissues and 144 normal mammary tissues, we collected the data of PGK1 (Reporter ID: ILMN_2216852) and G6PD (Reporter ID: ILMN_2347949) expression for analysis. Besides, we searched TCGA database targeting breast invasive carcinoma gene expression detected by RNA-seq (polyA + IlluminaHiSeq). Using the online TCGA analysis tool GEPIA (Gene Expression Profiling Interactive Analysis) [25], we compared the expression of PGK1 and G6PD between 1085 BC tissues and 112 normal mammary tissues. Gene expression data of the RNA-seq datasets were transformed to log_2_ (transcript count per million [TPM] + 1).

### Kaplan–Meier plotter database survival analysis

The prognostic role of PGK1 and G6PD in BC was assessed using the Kaplan–Meier plotter (http://www.kmplot.com) [26]. Kaplan–Meier plotter system includes gene chip and RNA-seq data (traced to GEO, EGA, and TCGA databases) involving survival information of over 54,000 genes in 21 cancer types. We evaluated the relevance of PGK1 and G6PD with RFS and overall survival (OS) in BC patients. Samples were divided into the high- and low- expressed groups according to the median level of the genes. The survival data of PGK1 (Probe ID: 200738_s_at) and G6PD (Probe ID: 202275_at) were exported for prognostic analysis.

### Patients and samples

This work involved 64 newly diagnosed BC patients in Nanfang Hospital, Southern Medical University (Guangzhou, China) from November 2016 to November 2017. Patient characteristics on tumor size, grading, clinical stage, and molecular pathology (ER/PR expression and HER-2 expression/amplification) are summarized in Additional file 1: Table S1. At baseline before any treatment, peripheral blood samples were collected from the patients for CTCs analysis. Next, patients were treated with neoadjuvant chemotherapy and/or surgical removal, with or without targeted therapy and endocrinotherapy, according to BC clinical guidelines. Patients were followed up regularly at the direction of physicians from the time of baseline CTCs test to the end time of the research. According to the *Response Evaluation Criteria in Solid Tumors* (RECIST) [27], disease progression with relapse or new metastasis, and death of any cause was recorded to assess the progression-free survival (PFS).

### CTCs detection and metabolic classification

CTCs enrichment and identification were performed using the Canpatrol system (SurExam, Guangzhou, China) based on micro-filtration, fluorescence staining and RNA in situ hybridization (ISH) methods, as previously described [20]. The blood sample (5 mL) was firstly treated with ammonium chloride buffer for erythrocyte lysis, followed by the membrane filtration step to eliminate leukocytes. The retained cells were treated with DAPI (Sigma, St. Louis, USA) for nuclear staining. Next, the labeled nucleic acid probes were added to hybridize with mRNA targets, including the Alexa Fluor 740-labeled CD45 probes and Alexa Fluor 647-labeled glucose metabolic (GM) markers (PGK1/G6PD). Through automatic microscopic scanning and imaging (Zeiss, Germany), the residual leukocytes were excluded by CD45 signal and the identified CTCs were divided into GM^+^ or GM^−^ subtype according to the expression of GM markers. Sequences of the capture probes for CD45, PGK1, and G6PD are shown in Additional file 2: Table S2.

### Determination of the positive criterion for CTCs parameters

The Youden Index was utilized to select the optimal cut-off for CTCs qualitative analysis. We simulated a receiver operating characteristic (ROC) curve for each CTCs parameter to assess the performance in the discrimination of cancer metastasis. The Youden Index was calculated by (sensitivity + specificity − 1), and the maximum was determined as the optimal cut-off value [28], which was set as the positive threshold of CTCs. The counting data of CTCs parameters could be qualitatively transformed into positive (≥ threshold) or negative (< threshold).

### Classification of the EMT phenotypes in CTCs

The EMT feature of identified CTCs was analyzed using the multi-RNA-ISH technology, according to the expression of epithelial (E) and mesenchymal (M) markers. Probes for E markers (EpCAM and CK8/18/19) and M markers (Vimentin and Twist) were labeled by Cy3 and Alexa Fluor 488 dyes, respectively. Capture probes for these markers were designed as shown in our previous report [29] and Additional file 3: Table S3. After the molecular hybridization and microscopic scanning, CTCs were classified as E-CTCs (epithelial type: E^+^M^−^), H-CTCs (hybrid type: E^+^M^+^) or M-CTCs (mesenchymal type: E^−^M^+^). In order to optimize the protocol of blood sample CTCs analysis, we integrated the detection of metabolic and EMT markers by simultaneously using the five fluorescence channels of the microscope system. The detected signals of Channel 1 to Channel 5 were DAPI, CD45, E markers, M markers and GM markers with different fluorescent labels (Additional file 4: Table S4). Besides the cell size-, nuclear morphology- and CD45-based screening, the enriched cells without any tumor markers (DAPI^+^CD45^−^E^−^M^−^) were further excluded in the CTCs identification. Next, the subtype of each CTC was analyzed by metabolic or EMT classification as described above.

### Statistical analysis

Data were presented by mean ± SD (continuous variables) and median with range or frequency distribution (discontinuous variables). Statistical analyses were performed using SPSS 19.0 (SPSS Inc., Chicago, USA), and the significant level was *P* < 0.05 (two-tailed test). Differences were compared through the Student’s *t* test or Mann–Whitney U test. The Chi square and Spearman’s rank correlation tests were used to evaluate the clinical relevance of CTCs. ROC curve and the area under the curve (AUC) were used to describe the diagnostic performance. Survival data were analyzed by Kaplan–Meier curve and Log-rank test. Hazard ratios (HR) and 95% CI (confidence intervals) were assessed by Cox regression analysis.

## Results

### Glucose metabolic classification of BC CTCs using PGK1/G6PD markers

**Fig. 1:**
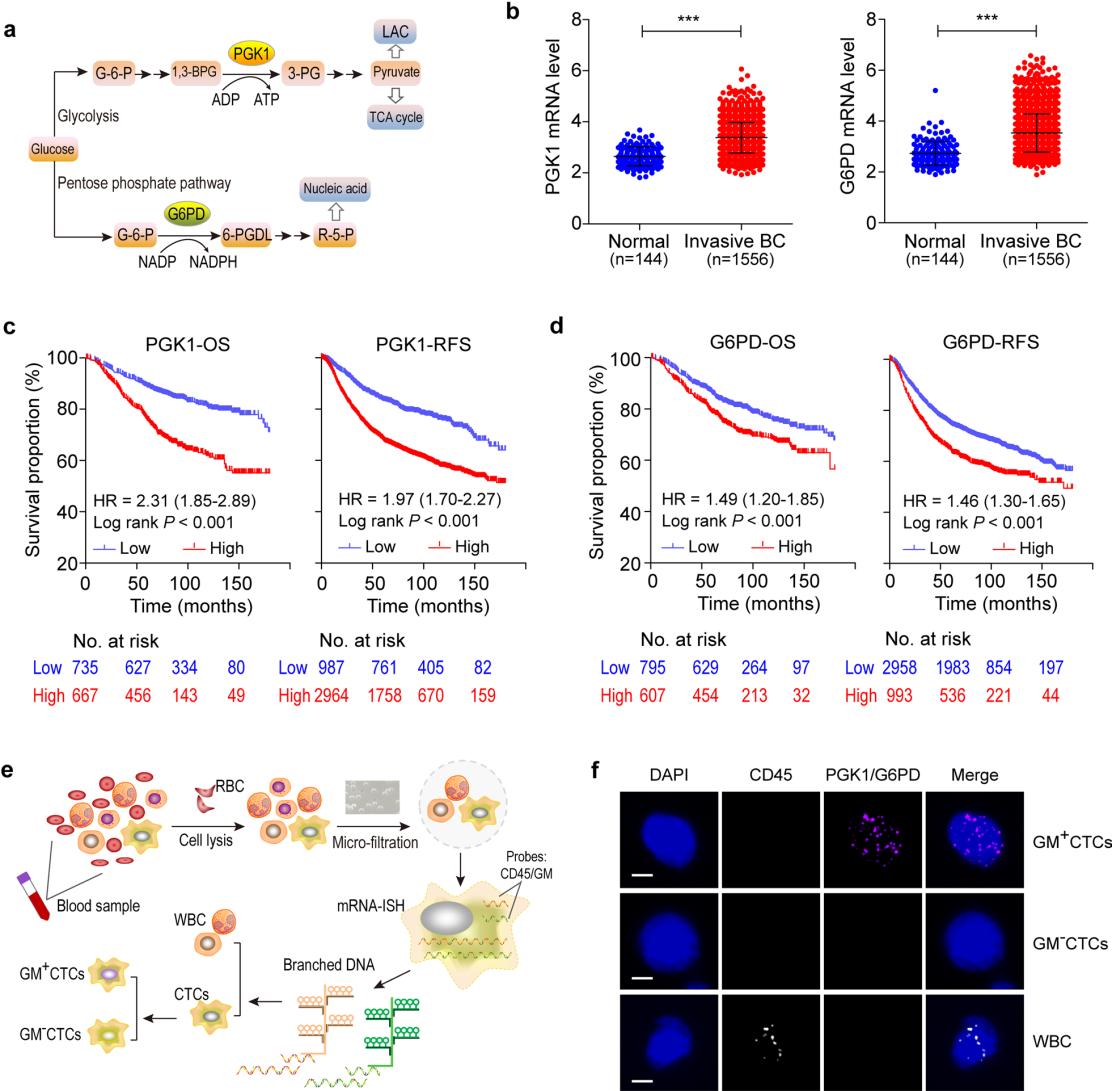
Glucose metabolic classification of CTCs using PGK1/G6PD markers. **a** The role of PGK1 and G6PD in glycolysis and the pentose phosphate pathway. **b** The mRNA level of PGK1 and G6PD in the tissues of normal mammary and invasive BC patients (Curtis Breast dataset from Oncomine database), ***P < 0.001. **c**, **d** Survival curves on overall and relapse-free survival in BC patients (Kaplan–Meier plotter database) who had high- versus low- PGK1 (**c**) and G6PD (**d**). **e** The workflow of CTCs enrichment, identification, and metabolic classification. **f** The fluorescence signal of DAPI, CD45, PGK1/G6PD, and merged pattern to characterize metabolic subtypes of CTCs. Scale bar = 5 μm. *G-6-P* glucose-6-phosphate, *1,3-BPG* 1,3-biphosphoglycerate, *3-PG* 3-phosphoglycerate, *LAC* lactic acid, *6-PGDL* 6-phosphogluconolactone, *R-5-P* ribose-5-phosphate

Previously we compared the metabolic gene expression between high and low metastatic cancer cells using microarray analysis, which screened 45 up-regulated genes in the high metastatic cancer cells [20]. Through a multi-factor weighted model enrolling the array results, cell validation, expression rates in CTCs and metastasis-related functions reported by literature, PGK1 and G6PD were determined the optimal metabolic markers related to tumor metastasis [20]. PGK1 gene encodes phosphoglycerate kinase to catalyze the reaction of 1,3-biphosphoglycerate to 3-phosphoglycerate; G6PD gene encodes glucose-6-phosphate dehydrogenase to convert glucose to ribose-5-phosphate (Fig. 1a). Before the markers were set for CTCs classification, we validated their relevance with BC using the Oncomine, TCGA and Kaplan–Meier plotter databases. Based on the Oncomine microarray data, the mRNA levels of PGK1 and G6PD were markedly increased in the invasive BC group (n = 1556) than that of the normal group (n = 144) (*P* < 0.001; Fig. 1b). Similar results were presented for TCGA RNA-seq data, demonstrating that PGK1 and G6PD were up-regulated in the invasive BC tissues (n = 1085) compared with the normal tissues (n = 112) (*P* < 0.05; Additional file 5: Figure S1). A total of 1402 and 3951 BC cases were involved in the analysis of OS and RFS, respectively (Fig. 1c, d). Median OS and RFS of the high-PGK1 patients were 70 and 61 months, shorter than that of the low-PGK1 patients (OS 96 months and RFS 90 months) (*P* < 0.001). Similarly, the high-G6PD patients presented inferior median OS and RFS than the low-G6PD patients (*P* < 0.001). These data indicated the increased tissue- PGK1 and G6PD could be a predictor for BC progression and prognosis.

The above results verified the availability of PGK1/G6PD as typing markers for CTCs. Here, we established an optimized operation flow for CTCs enrichment, identification, and metabolic classification (Fig. 1e). Mixed cells in the blood sample were differentiated through cell lysis, micro-filtration, mRNA-ISH, signal amplification, and microscopy analysis. CTCs were defined as large cells with a polymorphic nucleus, negative CD45 expression and positive expression of at least one tumor marker, whereas leukocytes were excluded for positive CD45 expression (Fig. 1f). Based on the expression of the combined PGK1/G6PD (GM) markers, the identified CTCs were further classified into metabolic subtypes. Cells labeled by DAPI^+^CD45^−^GM^+^ and DAPI^+^CD45^−^GM^−^ were respectively characterized as GM^+^CTCs and GM^−^CTCs (Fig. 1f).

### CTCs counting and metabolic subtypes indicate distant metastasis of BC

**Fig. 2:**
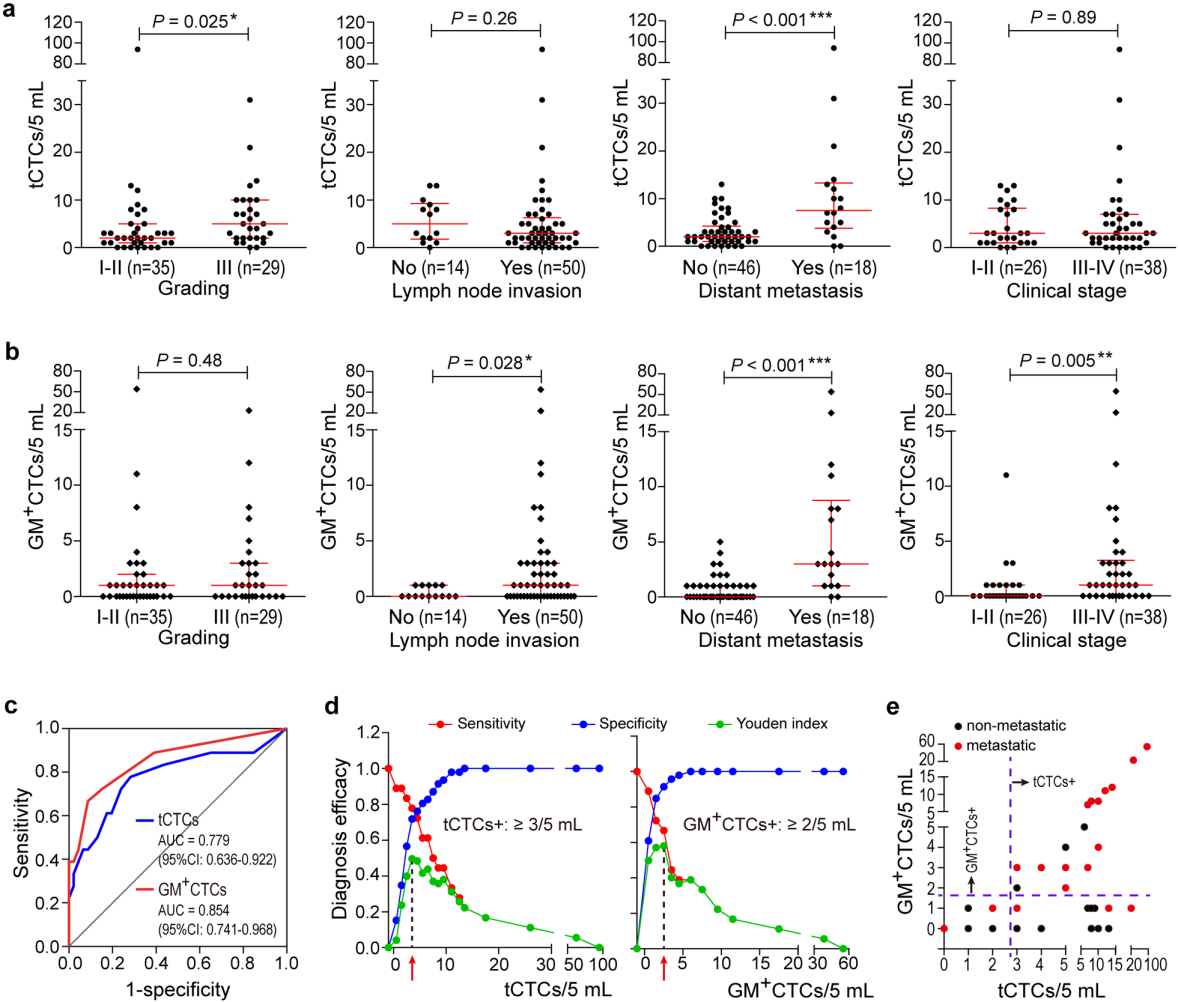
Correlation of tCTCs and GM^+^CTCs with the clinical feature of BC patients. **a**, **b** Cell numbers of tCTCs (**a**) and GM^+^CTCs (**b**) in clinical subgroups of grading, lymph node invasion, distant metastasis, and clinical stage. **c** ROC curves of tCTCs and GM^+^CTCs in the diagnosis of metastatic BC patients. **d** Determination of the positive threshold for tCTCs and GM^+^CTCs through the Youden index. **e** Comparison of the efficacy of tCTCs^+^ and GM^+^CTCs^+^ indexes in the diagnosis of BC metastasis. *P < 0.05, **P < 0.01 and ***P < 0.001

To explore the clinical significance of CTCs, we detected the total CTCs (tCTCs) and CTCs metabolic phenotypes of 64 BC patients. Patient characteristics are summarized in Additional file 1: Table S1, including age, tumor size, grading, disease stage, metastasis and molecular pathology. We found detectable tCTCs in 55 out of 64 patients (85.9%), with a range of 0–94 cells in 5 mL of blood. In the group comparisons (Fig. 2a), the grading III group showed higher level of tCTCs than the grading I-II group (*P* = 0.025). The median tCTCs number of metastatic group (8 cells/5 mL) was markedly increased than that of non-metastatic group (2 cells/5 mL; *P* < 0.001). No obvious difference of tCTCs number was observed between different lymph node status groups (*P* = 0.26) or clinical stage groups (*P* = 0.89). GM^+^CTCs was detectable in 61.8% (34/55) of tCTCs > 0 patients with a range of 0–54 cells/5 mL. GM^+^CTCs level was obviously increased in metastatic group compared with non-metastatic group (*P* < 0.001; Fig. 2b). Besides, differences of GM^+^CTCs were significant between the lymph node status groups (*P* = 0.028) or clinical stage groups (*P* = 0.005) but not the grading groups (*P* > 0.05; Fig. 2b). These data indicated the correlation of tCTCs and GM^+^CTCs with BC metastasis. Moreover, ROC curves represented the AUC was 0.779 (95% CI 0.636–0.922) for tCTCs and 0.854 (95% CI 0.741–0.968) for GM^+^CTCs in the diagnosis of BC metastasis (Fig. 2c).

Furthermore, we compared the positive rates of tCTCs and GM^+^CTCs between BC patients with different clinical characteristics. Since there is currently no uniform criterion for positive/negative CTCs, we used the Youden Index (= sensitivity + specificity-1) [27] to determine the positive threshold in this work. The maximum Youden Index pointed to 3/5 mL for the tCTCs count and 2/5 mL for GM^+^CTCs count, respectively (Fig. 2d). Accordingly, patients got a tCTCs^+^ report if tCTCs ≥ 3/5 mL, and a GM^+^CTCs^+^ report if GM^+^CTCs ≥ 2/5 mL. The tCTCs^+^ rate was remarkably correlated with distant metastasis, ER expression and PR expression (*P* < 0.05; Table 1). The GM^+^CTCs^+^ rate was correlated with lymph node invasion, distant metastasis, and clinical stage (*P* < 0.05; Table 1). No significant relevance was found between the positive rate of tCTCs or GM^+^CTCs and other features. These data validated the indicative role of tCTCs and GM^+^CTCs in BC metastasis. The sensitivity and specificity of the tCTCs^+^ index were 77.8% and 71.8% in the diagnosis of metastasis. For the GM^+^CTCs^+^ index, the sensitivity and specificity were 66.7% and 91.3% (Fig. 2d, e).

**Table 1:** Clinical feature of BC patients and the correlation with tCTCs and GM^+^CTCs

Clinical characteristics		tCTCs		GM^+^CTCs	
Subgroup	n	P/N^a^	*P*	P/N^b^	*P*
Age (years)			0.138		0.147
≤ 50	31	14/17		7/24	
> 50	33	21/12		13/20	
Histology			0.475		0.454
Ductal	52	29/23		16/36	
Lobular	6	2/4		1/5	
Other	6	4/2		3/3	
Tumor size			0.946		0.194
≤ 5 cm	51	28/23		14/37	
> 5 cm	13	7/6		6/7	
Grading			0.113		0.294
I–II	35	16/19		9/26	
III	29	19/10		11/18	
Lymph node invasion			0.414		0.011*
No	14	9/5		0/14	
Yes	50	26/24		20/30	
Distant metastasis			0.004*		< 0.001*
No	46	20/26		7/39	
Yes	18	15/3		13/5	
Clinical Stage			0.911		0.005*
I–II	26	14/12		3/23	
III–IV	38	21/17		17/21	
ER expression			0.036*		0.076
–	22	16/6		10/12	
+	42	19/23		10/32	
PR expression			0.026*		0.227
–	25	18/7		10/15	
+	39	17/22		10/29	
HER2 expression			0.469		0.533
–	16	10/6		6/10	
+	48	25/23		14/34	
HER2 amplification			0.729		0.917
–	39	22/17		12/27	
+	25	13/12		8/17	

^a^*P* positive, *N* negative. The positive criterion of tCTCs is ≥ 3/5 mL

^b^*P* positive, *N* negative. The positive criterion of GM^+^CTCs is ≥ 2/5 mL

*P < 0.05

### Comparison of EMT and metabolic CTCs subtypes in metastasis diagnosis

**Fig. 3:**
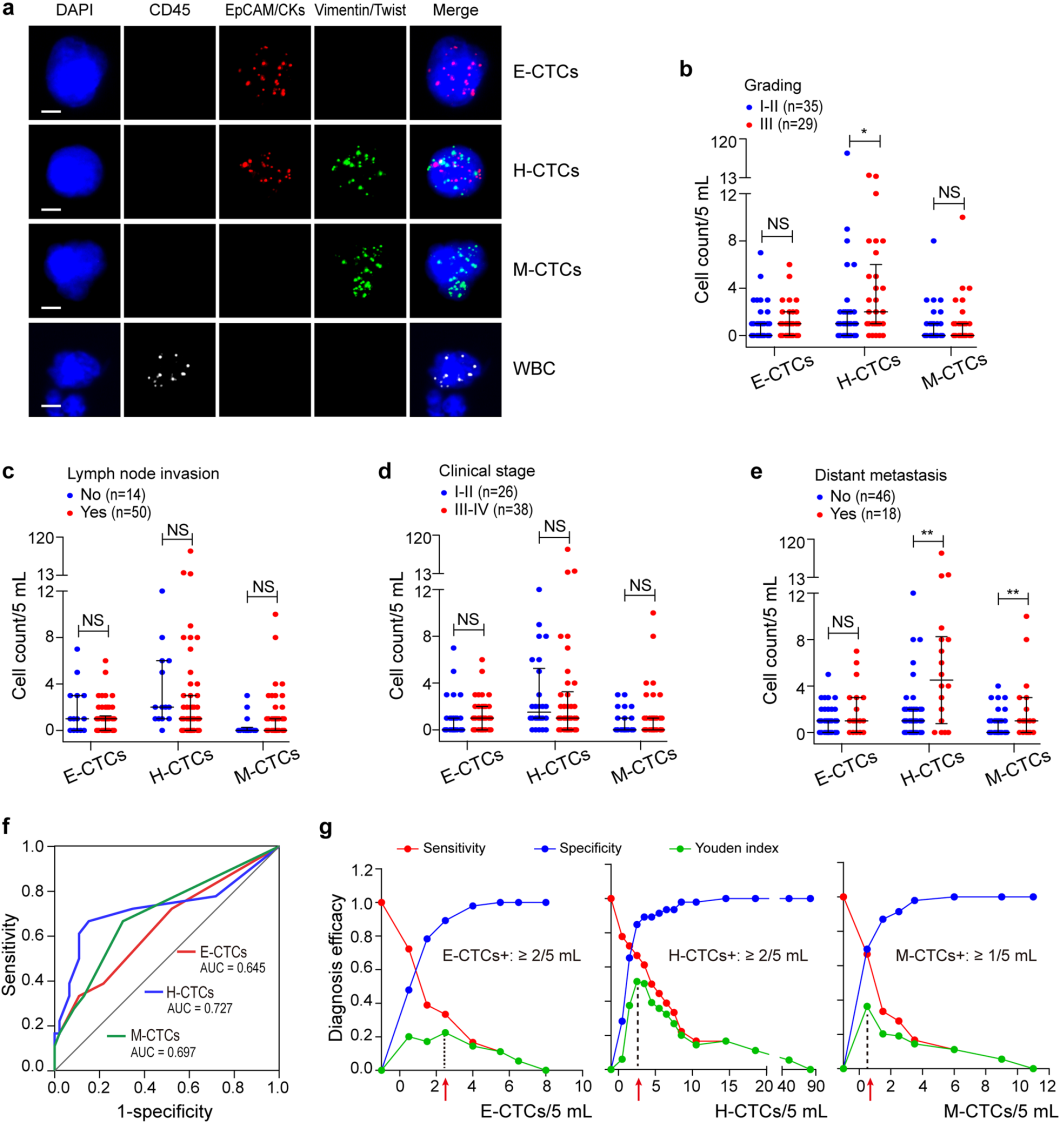
The EMT phenotypes of CTCs and their correlations with BC patient feature. **a** Images of the fluorescence signal of DAPI, CD45, EpCAM/CKs, Vimentin/Twist and merged pattern to characterize the EMT phenotypes of CTCs. Scale bar = 5 μm. **b**–**e** Comparison of E-CTCs, H-CTCs, and M-CTCs numbers between clinical subgroups of grading (**b**), lymph node invasion (**c**), clinical stage (**d**) and distant metastasis (**e**). **f** ROC curves of E-CTCs, H-CTCs, and M-CTCs in the diagnosis of metastatic BC patients. **g** Determination of positive threshold for E-CTCs, H-CTCs, and M-CTCs through the Youden index. *P < 0.05, **P < 0.01 and NS means not significant (*P* > 0.05)

We profiled the EMT phenotypes of CTCs through the epithelial (EpCAM/CKs) and mesenchymal (Vimentin/Twist) markers (Fig. 3a). Totally 67.3%, 85.5% and 47.3% of the tCTCs > 0 patients had detectable E-CTCs, H-CTCs and M-CTCs, with a range of 0–7, 0–81 and 0–10 cells/5 mL. The level of H-CTCs but not E-CTCs or M-CTCs was higher in grading III patients versus grading I-II patients (*P* = 0.045; Fig. 3b). There was no obvious difference in the number of three subtypes between the lymph node status groups or clinical stage groups (*P* > 0.05; Fig. 3c, d). However, metastatic group presented an increased level of H-CTCs and M-CTCs compared with non-metastatic group (*P* < 0.01; Fig. 3e). The AUCs of ROC curves of E-CTCs, H-CTCs and M-CTCs were 0.645 (0.488–0.802), 0.727 (0.562–0.892) and 0.697 (0.548–0.847) for metastasis diagnosis (Fig. 3f). Next, the Youden Index curves determined the positive threshold as E-CTCs ≥ 2/5 mL, H-CTCs ≥ 2/5 mL, and M-CTCs ≥ 1/5 mL (Fig. 3g). The H-CTCs^+^ rate and M-CTCs^+^ rate were closely correlated with distant metastasis (Additional file 6: Table S5; *P* < 0.01). No significant correlation was found between E-CTCs^+^ rate and the pathological characteristics (Additional file 6: Table S5). These data highlighted the indicative role of H-CTCs and M-CTCs for BC metastasis.

**Fig. 4:**
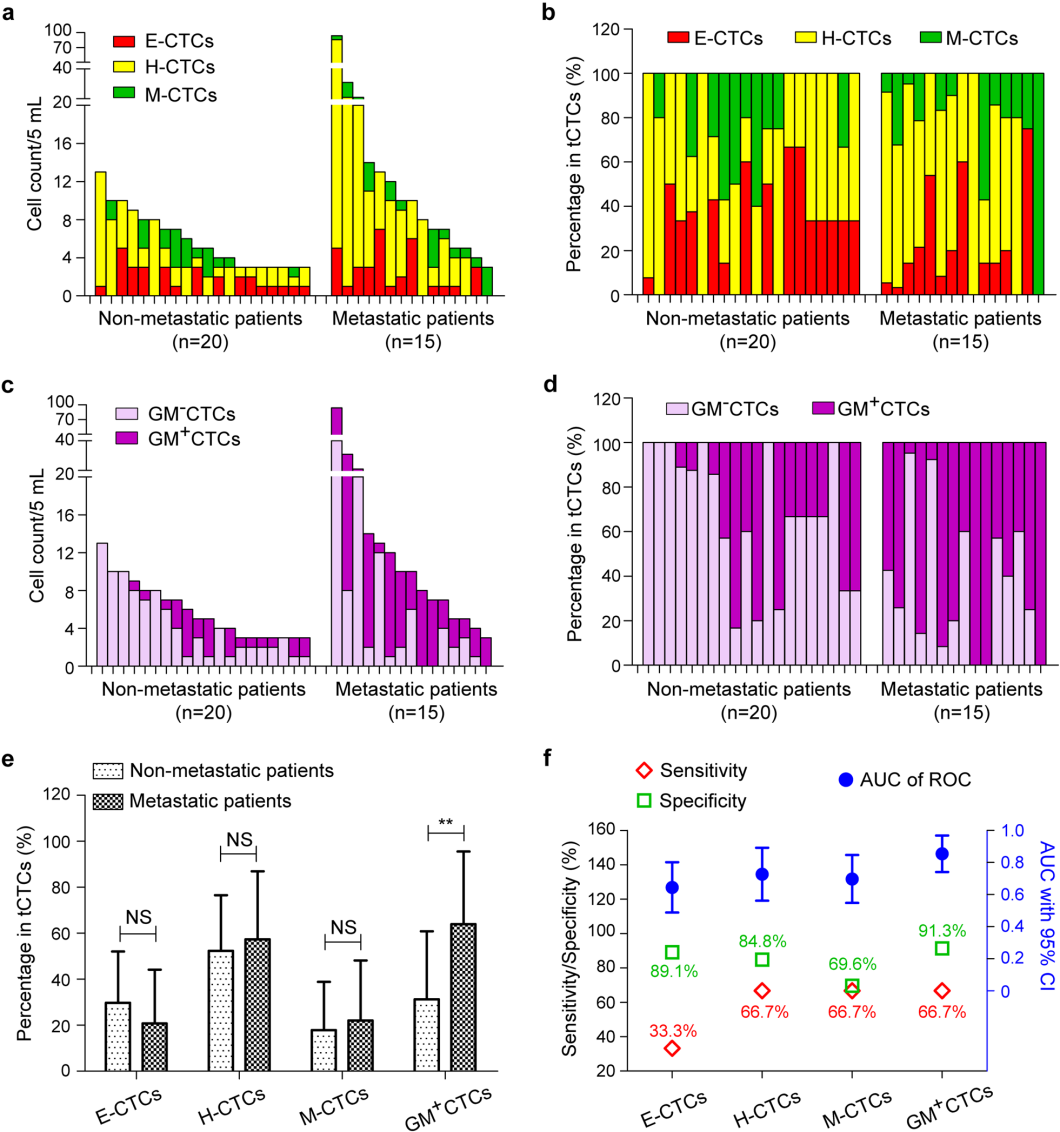
The EMT/metabolic CTCs subtypes in metastatic and non-metastatic BC patients. **a**, **b** Cell number and proportion (in tCTCs) of the EMT subtypes (E-CTCs, H-CTCs, and M-CTCs) in metastatic and non-metastatic groups. **c**, **d** Cell number and proportion (in tCTCs) of the metabolic subtypes (GM^+^CTCs and GM^−^CTCs) in metastatic and non-metastatic groups. **e** Comparison of CTCs subtype proportions between the metastatic and non-metastatic group. **P < 0.01 and NS means not significant (*P* > 0.05). **f** The diagnostic performance (AUCs and sensitivity/specificity) of CTCs subtypes in distinguishing cancer metastasis

To compare the distribution of EMT/metabolic CTCs subtypes between metastatic and non-metastatic BC patients, we analyzed the percentage of each subtype in tCTCs. Cases that had positive tCTCs (≥ 3/5 mL) were included in this analysis, including 15 metastatic patients and 20 non-metastatic patients (Fig. 4a–d). In the EMT classification, except the major H-CTCs in both groups (average 52.4% and 57.3% of tCTCs in non-metastatic and metastatic group), E-CTCs accounted for average 29.7% of tCTCs in non-metastatic group whereas M-CTCs accounted for average 22.0% of tCTCs in metastatic group (Fig. 4a, b). In metabolic classification, the average proportion of GM^+^CTCs in tCTCs was 64.0% in metastatic group and 31.3% in non-metastatic group (Fig. 4c, d). The difference of subtype percentages was marked for GM^+^CTCs (*P* = 0.004) but not significant for the EMT types (*P *> 0.05; Fig. 4e). Overall, in the diagnosis of BC metastasis, the AUCs of CTCs subtypes ranked as GM^+^CTCs (0.854), H-CTCs (0.727), M-CTCs (0.697) and E-CTCs (0.645). With the same sensitivity of 66.7%, GM^+^CTCs showed better specificity (91.3%) than H-CTCs (84.8%) and M-CTCs (69.6%) (Fig. 4f).

### CTCs subtypes are prognostic markers of survival in BC patients

**Fig. 5:**
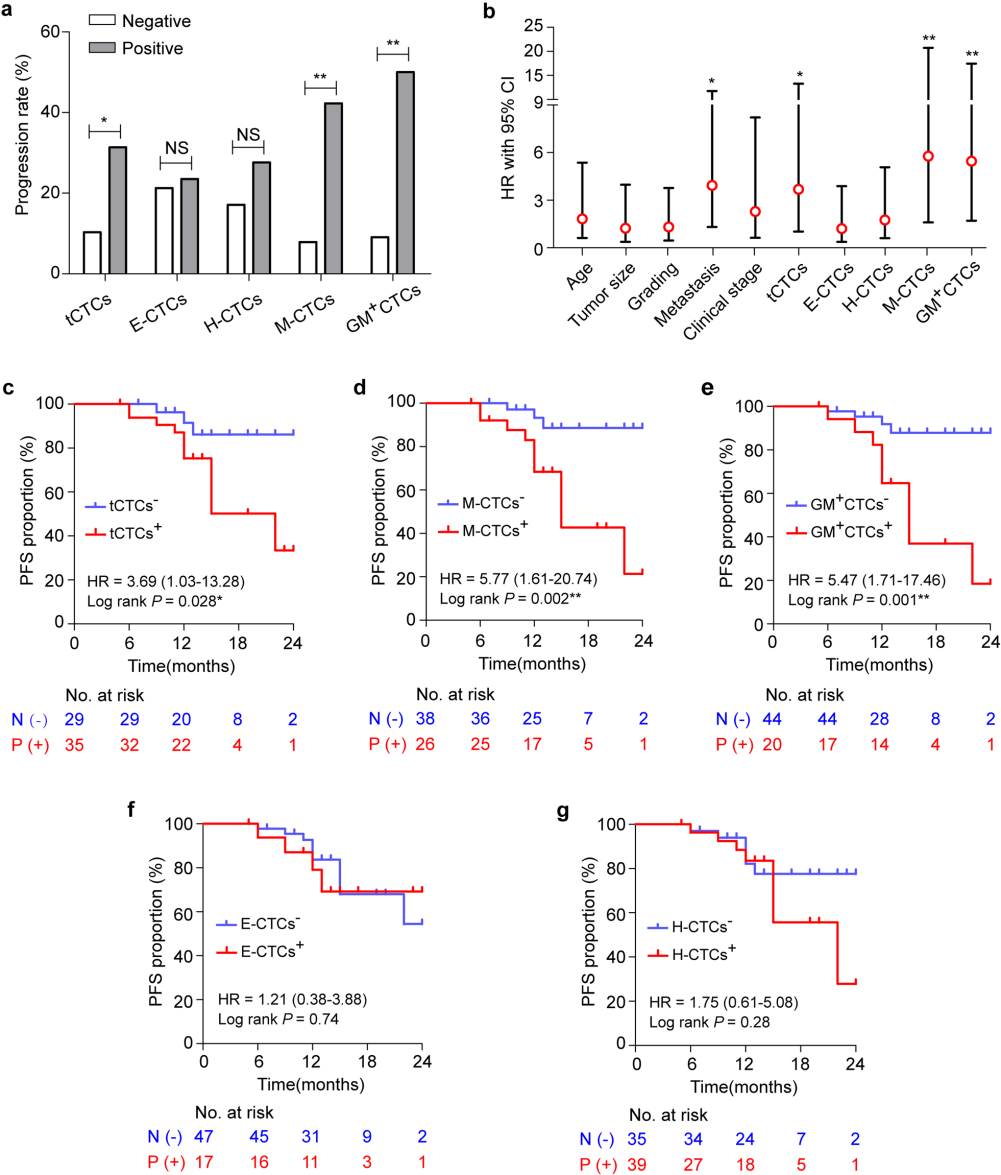
Relevance of CTCs test with the progression-free survival (PFS) of BC patients. **a** Comparison of disease progression ratios between patients with positive and negative CTCs test (tCTCs, E-CTCs, H-CTCs, M-CTCs, and GM^+^CTCs). **b** The Hazard ratio (HR) with 95% CI of the CTCs parameters and pathological characteristics for the PFS of BC patients based on Cox regression analysis. **c**–**g** Kaplan–Meier plot analysis for PFS according to different levels of tCTCs (**c**), M-CTCs (**d**), GM^+^CTCs (**e**), E-CTCs (**f**) and H-CTCs (**g**). *P < 0.05, **P < 0.01 and NS means not significant (*P* > 0.05)

To evaluate the CTCs parameters as a predictor for cancer prognosis, we studied their relevance with disease progression and PFS in the 64 BC patients. The median follow-up time was 13 (range 5–24) months starting from baseline CTCs test. A progression event occurred when patients had progressive disease (according to RECIST), relapse or new metastasis, and death of any cause. Progression rates of tCTCs^+^ patients and tCTCs^−^ patients were 31.4% and 10.3% (*P* = 0.042; Fig. 5a). No significant difference was observed between the progression rates of E-CTCs^+^ versus E-CTCs^−^ groups or H-CTCs^+^ versus H-CTCs^−^ groups (*P* > 0.05; Fig. 5a and Additional file 7: Table S6). However, the M-CTCs^+^ and GM^+^CTCs^+^ groups showed increased progression rates at 42.3% and 50.0% compared with M-CTCs^−^ (7.9%) and GM^+^CTCs^−^ (9.1%) groups (*P* < 0.01; Fig. 5a). The cox regression analysis revealed that patients with baseline positive CTCs had inferior PFS (Fig. 5b and Table 2), including tCTCs (HR = 3.69; *P *= 0.046), M-CTCs (HR = 5.77; *P* = 0.007) and GM^+^CTCs (HR = 5.47; *P* = 0.004). The PFS proportion of the tCTCs^+^ group was remarkably decreased than that of the tCTCs^−^ group at 2 years (*P* = 0.028; Fig. 5c). In comparison of the M-CTCs^−^ and M-CTCs^+^ groups, the PFS proportions were 88.6% versus 21.4% at 2 years (*P* = 0.002; Fig. 5d). In GM^+^CTCs^−^ and GM^+^CTCs^+^ groups, the proportions were 87.9% versus 18.5% at 2 years (*P* = 0.001; Fig. 5e). No remarkable difference was observed in the PFS proportions between the E-CTCs^+^ versus E-CTCs^−^ or H-CTCs^+^ versus H-CTCs^−^ groups (*P* > 0.05; Fig. 5f, g). These data proved the significance of tCTCs, M-CTCs, and GM^+^CTCs to be prognostic markers of BC patients.

**Table 2:** Cox regression analysis of PFS in BC patients

Variables	Hazard ratio	95% CI	*P*^a^
Age, years (> 50 vs. ≤ 50)	1.83	0.62–5.37	0.27
Tumor size, cm (> 5 vs. ≤ 5)	1.23	0.38–3.98	0.73
Grading (III vs. I–II)	1.32	0.46–3.77	0.61
Distant metastasis (yes vs. no)	3.93	1.31–11.77	0.014*
Clinical stage (III–IV vs. I–II)	2.28	0.64–8.22	0.21
tCTCs (P vs. N)^b^	3.69	1.03–13.28	0.046*
E-CTCs (P vs. N)	1.21	0.38–3.88	0.74
H-CTCs (P vs. N)	1.75	0.61–5.08	0.30
M-CTCs (P vs. N)	5.77	1.61–20.74	0.007**
GM^+^CTCs (P vs. N)	5.47	1.71–17.46	0.004**

^a^* *P* < 0.05, **P < 0.01

^b^*P* positive, *N* negative

## Discussion

The predictive and prognostic roles of CTCs have been recently investigated in the BC setting. However, the heterogeneity of CTCs remains a challenge for the clinical application of CTCs test. Here, we used PGK1/G6PD markers to design a metabolic typing method for CTCs by integrating the membrane filtration, fluorescent staining, and multi-mRNA-ISH techniques. We determined the positive/negative criteria for the CTCs metabolic subtypes and evaluated their clinical relevance in BC patients. Comparisons between EMT and metabolic subtypes of CTCs were performed to assess their significance in predicting BC metastasis and prognosis.

Metabolic reprogramming is a hallmark of cancer cells involved in the promotion of tumorigenesis and progression [30]. PGK1 and G6PD, as crucial metabolic markers of glycolysis and the pentose phosphate pathway, have been demonstrated to favor metastasis of various cancers [31–35]. In vitro and in vivo studies showed that knockdown of PGK1 reduced proliferation and metastasis of SNU449 and HCCLM3 cells, indicating a driving role of PGK1 in hepatocellular carcinoma progression [31]. Similar studies from Yu et al. [32] and Ahmad et al. [33] presented that PGK1 enhanced metastasis of lung and colon cancer through the activated AKT/mTOR pathway and JUN/FOS pathway. Overexpression of G6PD could regulate the Notch1/HES-1 pathway in MCF-7 and MDA-MB-231 cells to promote BC metastasis [34]. Mele et al. [35] used Polydatin (100 mg/kg) to block G6PD and the pentose phosphate pathway and observed about 80% inhibition of lymph node metastases in a metastatic mouse model. In the present work, we found PGK1 and G6PD were markedly up-regulated in invasive BC tissues compared with normal mammary tissues and indicated shorter RFS and OS of BC patients. The data were parallel with previous clinical researches, which demonstrated PGK1 and G6PD could predict a high risk of recurrent metastasis and poor prognosis in BC [22, 36]. These results confirmed the significance of PGK1 and G6PD in BC metastasis and indicated their feasibility for CTCs metabolic classification.

The optimized operation flow for CTCs analysis used in this work facilitates synchronous identification and classification of CTCs. This system presented favorable sensitivity for CTCs capture representing a prevalence of 85.9% in BC patients, which was better than reports on the FDA-approved CellSearch method in BC (38.0–56.7%) [37, 38]. Moreover, leukocytes were eliminated by firstly size-based filtration and secondly CD45 marker recognition to guarantee the purity of CTCs, equipping the system with higher specificity than the size-based or negative-enriched methods alone. We found the increased tCTCs number was markedly associated with distant metastasis and unfavorable pathological grading, suggesting the importance of CTCs in promoting metastasis and disease progression. In the updated TNM staging for BC, the NCCN guidelines have added a new M0 (i +) category which is defined as “no clinical or radiographic evidence of distant metastases, but deposits of detected tumor cells in the circulation fluids” [39]. Remarkably, in spite of the low proportion of GM^+^CTCs in tCTCs (median 9.4%), GM^+^CTCs presented close relevance with metastasis as well as lymph node status and clinical stage. These results provided support for Yu’s [7] research which demonstrated only a small part of CTCs could finally form metastases and suggested the vital role of GM^+^CTCs in promoting metastasis and disease progression.

Although various methods have been developed for CTCs detection, there is a lack of uniform cut-off value for clinical utility. Most CellSearch studies set the standard at 5 CTCs/7.5 mL for BC ever since Cristofanilli’s group demonstrated that the baseline level of ≥ 5 CTCs/7.5 mL was associated with shorter PFS and OS [40, 41]. Reported standards for other methods include ≥ 1, ≥ 2, and ≥ 3 CTCs in 5–7.5 mL of blood sample [20, 40, 42]. It is reasonable to define different positive criteria of CTCs for diverse methods since the compositions of captured CTCs are not the same depending on the enrichment principle. Here, we determined the positive threshold of CTCs parameters using the Youden Index curve, which could suggest the best cut-off value by fitting optimal sensitivity and specificity [28]. By the threshold of tCTCs ≥ 3/5 mL and GM^+^CTCs ≥ 2/5 mL, we observed favorable performance of GM^+^CTCs and tCTCs in the diagnosis of BC metastasis. Though tCTCs presented higher sensitivity than GM^+^CTCs (77.8% versus 66.7%), GM^+^CTCs presented higher specificity than tCTCs (91.3% versus 71.8%). The data verified our hypothesis that metabolically active CTCs might represent the aggressive CTCs subpopulations in cancer metastasis. Thus GM^+^CTCs turned out to be a more specific marker than tCTCs. This phenomenon could also account for the inconsistent results that advanced clinical stage and lymph node status were relevant to increased GM^+^CTCs but not tCTCs. Since only a small part of the CTCs are functionally active [7, 8], the non-aggressive CTCs such GM^−^CTCs may be confounding factors to affect the correlation analysis. Overall, these results indicated GM^+^CTCs could be a powerful marker of BC metastasis as a supplement for the tCTCs test.

Ever since the existence of EMT in CTCs was highlighted [9], several studies have profiled the EMT phenotypes of CTCs in primary and metastatic BC [43–45]. For instance, Mego’s group evaluated the expression of EMT transcription factors (Twist, Snail1, Slug, and Zeb1) in CTCs by RT-PCR in 427 primary BC patients and found EMT-CTCs were associated with inferior prognosis [43]. Results of the present work were in concordance with the above reports and our early studies on hepatocellular carcinoma and prostate cancer [11, 20]. We found that BC metastasis was significantly correlated with increased H-CTCs and M-CTCs but not E-CTCs, and H-CTCs presented the best diagnosis performance among the three subtypes. Although the AUCs of EMT subtypes (0.645–0.727) was lower than that of GM^+^CTCs (0.854), H-CTCs showed a favorable specificity of 84.8% (tCTCs 71.8% and GM^+^CTCs 91.3%) in the metastasis diagnosis. This result also gives evidence for recent research by Liu’s group [45]. They found epithelial-type CTCs with a restricted mesenchymal transition had the most potent ability of lung metastases formation in a BC mouse model. The hybrid CTCs (E^+^M^+^) might represent the most plastic cells in cancer metastasis [46].

Concomitant metabolic reprogramming is induced during the activation of EMT and cancer metastasis. Compared with epithelial cells, mesenchymal tumor cells have different metabolic requirements to meet the increased energy demands for migration and invasion [47]. Shaul’s group designed a mesenchymal-like metabolic gene expression signature by the database analysis on 978 human cancer cell lines. This signature includes 44 metabolic genes that are essential for EMT but not cell proliferation, involving metabolism regulation of glucose, lipid, and nucleotide [48]. Noticeably, functional studies demonstrated that knockdown of PGK1 could reverse the EMT process to inhibit invasion of BC cells [21]. An investigation on hepatocellular carcinoma indicated that G6PD could activate the STAT3 pathway to promote EMT and further favor cancer metastasis [49]. Consistent with the above studies, we observed an association of GM^+^CTCs number with H-CTCs (R^2^ = 0.852, *P* < 0.001) and M-CTCs (R^2^ = 0.591, *P* < 0.001) numbers, whereas no obvious correlation was observed between GM^+^CTCs and E-CTCs (*P* = 0.10; Additional file 8: Figure S2). Moreover, the proportions of GM^+^CTCs in H-CTCs (51.8%) and M-CTCs (47.5%) were remarkably higher than those in E-CTCs (27.6%; Additional file 8: Figure S2). These results demonstrated an intensive correlation between CTCs metabolic subtypes and EMT subtypes. The two classifications of CTCs, representing respectively the functional and morphological features of heterogeneous CTCs, could synergistically enrich the significance of CTCs test.

Metastasis is the leading cause of recurrence and tumor-related death. Given the driving role of CTCs in cancer metastasis, we investigated the prognostic role of CTCs parameters. Positive tCTCs, M-CTCs, and GM^+^CTCs presented a predictive function of increased progression ratio and decreased 2 years PFS ratio. Previous studies on the prognosis value of EMT CTCs showed controversial conclusions [43, 50, 51]. Apart from the dynamic changes of EMT status occurred in CTCs dissemination, reasons for the conflicts include different detection methods involved in these researches and the lack of a uniform cut-off standard. Our study indicated GM^+^CTCs to be a potential predictor for inferior prognosis in BC patients. In addition, the analysis of TCGA data revealed the up-regulation of PGK1 and G6PD in common cancers such as colon, lung and gastric cancers (Additional file 9: Figure S3). These results suggest the possibility of applying the PGK1/G6PD-based CTCs metabolic classification method in other cancer diseases besides BC, though further validation remains in need before practical applications.

One shortcoming of this study is the limited follow-up time (maximum 24 months), which results in the immaturity of overall survival investigation. Long term observational studies on this cohort as well as an expanded sample size are underway to explore the prognostic value of CTCs subtypes. Furthermore, direct detection of the metabolic phenotypic features of the CTCs subpopulation is needed in future research to validate the active metabolic level of GM^+^CTCs. The emerging techniques such as microfluidic chip, nanomaterials and AIEgens (luminogens with aggregation-induced emission) might provide vital ideas for convenient CTCs analysis methods which target on glucose uptake, oxygen consumption, lactate production, ATP synthesis or other key regulators of metabolism. With the technical development of single-cell sequencing, multi-omics analysis and three-dimensional cell culture, the in vivo and in vitro mechanism investigations on GM^+^CTCs are desirable to illustrate the CTCs-related metabolic reprogramming, which could further enrich the metastasis theory and promote the application of CTCs classification.

## Conclusion

This work establishes a PGK1/G6PD-based method for CTCs glucose metabolic (GM) classification and assesses the clinical value of CTCs metabolic subtypes in BC. Hypermetabolic CTCs (GM^+^CTCs) marked by PGK/G6PD^+^ are promising biomarkers for metastasis diagnosis and prognosis prediction in BC. A positive level of baseline GM^+^CTCs (≥ 2/5 mL) indicates distant metastasis with a sensitivity of 66.7% and specificity of 91.3%, as well as an inferior PSF ratio of BC patients. The metabolic classification of CTCs provides clues for the identification of aggressive CTCs subpopulation and the development of new targeted drugs for cancer patients.

## Supplementary information

**Additional file 1: Table S1.** Clinical characteristics of the investigated BC patients (n = 64).

**Additional file 2: Table S2.** Probes of CD45, PGK1 and G6PD genes used in RNA-ISH [20].

**Additional file 3: Table S3.** Capture probes of the EMT markers used in RNA-ISH [29].

**Additional file 4: Table S4.** Parameters of the fluorescent channels in practical CTCs analysis

**Additional file 5: Figure S1.** The mRNA expressions of PGK1 and G6PD in TCGA breast invasive cancer cohort.

**Additional file 6: Table S5.** Correlation between CTCs EMT subtypes and clinical data of BC patients.

**Additional file 7: Table S6.** Correlation between CTCs parameters and disease progression of BC patients.

**Additional file 8: Figure S2.** Correlation between the EMT and metabolic subtypes of CTCs in BC patients.

**Additional file 9: Figure S3.** The mRNA expressions of PGK1 and G6PD in common cancers based on TCGA RNA-seq data.

## Data Availability

All data generated or analyzed during this study are included in the article and its additional files.

## Abbreviations

CTCs: Circulating tumor cells
BC: Breast cancer
EMT: Epithelial-mesenchymal transition
PGK1: Phosphoglycerate kinase 1
G6PD: glucose-6-phosphate dehydrogenase
RFS: Relapse-free survival
ISH: In situ hybridizatio
GM: Glucose metabolic
ROC: Receiver operating characteristic
PFS: Progression-free survival
OS: Overall survival
AUC: Area under the curve
HR: Hazard ratios
CI: Confidence intervals
