# Supplementary material for: Metabolic classification of circulating tumor cells as a biomarker for metastasis and prognosis in breast cancer
Source: J Transl Med. 2020 Feb 6;18:59. doi: 10.1186/s12967-020-02237-8 (PMC7003411; doi:10.1186/s12967-020-02237-8)
Supplement: Supplementary file 1 — Additional file 1: Table S1. Clinical characteristics of the investigated BC patients (n = 64). [file 12967_2020_2237_MOESM1_ESM.docx]

**Additional file 1:**

**Table S1 Clinical characteristics of the investigated BC patients (n = 64)**

| **Characteristics** | **n** | **Percentage** | **Average (Range)** |
| --- | --- | --- | --- |
| Age (years) |  |  | 50 (28-73) |
| ≤ 50 | 31 | 48.4% |  |
| > 50 | 33 | 51.6% |  |
| Histology |  |  | NA^a^ |
| Ductal | 52 | 81.2% |  |
| Lobular | 6 | 9.4% |  |
| Other | 6 | 9.4% |  |
| Tumor size |  |  | 3.7 (0.9-10.0) |
| ≤ 5cm | 51 | 79.7% |  |
| > 5cm | 13 | 20.3% |  |
| Grading |  |  | NA |
| I-II | 35 | 54.7% |  |
| III | 29 | 45.3% |  |
| Lymph node invasion |  |  | NA |
| No | 14 | 21.9% |  |
| Yes | 50 | 78.1% |  |
| Distant metastasis |  |  | NA |
| No | 46 | 71.9% |  |
| Yes | 18 | 28.1% |  |
| Clinical Stage |  |  | NA |
| I-II | 26 | 40.6% |  |
| III-IV | 38 | 59.4% |  |
| ER expression |  |  | NA |
| - | 22 | 34.4% |  |
| + | 42 | 65.6% |  |
| PR expression |  |  | NA |
| - | 25 | 39.1% |  |
| + | 39 | 60.9% |  |
| HER2 expression |  |  | NA |
| - | 16 | 25.0% |  |
| + | 48 | 75.0% |  |
| HER2 amplification |  |  | NA |
| - | 39 | 60.9% |  |
| + | 25 | 39.1% |  |

a, NA: not available.
