# Supplementary material for: Metabolic classification of circulating tumor cells as a biomarker for metastasis and prognosis in breast cancer
Source: J Transl Med. 2020 Feb 6;18:59. doi: 10.1186/s12967-020-02237-8 (PMC7003411; doi:10.1186/s12967-020-02237-8)
Supplement: Supplementary file 2 — Additional file 2: Table S2. Probes of CD45, PGK1 and G6PD genes used in RNA-ISH [20]. [file 12967_2020_2237_MOESM2_ESM.docx]

**Additional file 2:**

**Table S2 Probes of CD45, PGK1 and G6PD genes used in RNA-ISH [20]**

| **Gene** | **Probe Sequences (5’-3’)** |
| --- | --- |
| CD45 (Leukocyte marker) | TCGCAATTCTTATGCGACTC  TGTCATGGAGACAGTCATGT  GTATTTCCAGCTTCAACTTC  CCATCAATATAGCTGGCATT  TTGTGCAGCAATGTATTTCC  TACTTGAACCATCAGGCATC |
| PGK1 (Glucose metabolic marker) | TTTAACGTCCAGCTTGTCCA  AGTCGACTCTCATAACGACC  TCTGGTTGTTCTTCATAGGA  TTAATCCTCTGGTTGTTTGT  AATTTGATGCTTGGGACAGC  ACTTGGCTCCATTGTCCAAG  AGGTGGCTCATAAGGACTAC  TACTTGTCAGGCATGGGCAC |
| G6PD (Glucose metabolic marker) | GAAGTGTACGACCGTTTCCG  AAAAGCTCTTCCCGCAGGAT  CGACTGATGGAAGGCATCGC  CACCAGATGGTGGGGTAGAT  ACGATGAAGGTGTTTTCGGG  AGGAGTTGCGGGCAAAGAAG  TAGGAGGCTGCATCATCGTA  CATTCATGTGGCTGTTGAGG |
