# Supplementary material for: Metabolic classification of circulating tumor cells as a biomarker for metastasis and prognosis in breast cancer
Source: J Transl Med. 2020 Feb 6;18:59. doi: 10.1186/s12967-020-02237-8 (PMC7003411; doi:10.1186/s12967-020-02237-8)
Supplement: Supplementary file 3 — Additional file 3: Table S3. Capture probes of the EMT markers used in RNA-ISH [29]. [file 12967_2020_2237_MOESM3_ESM.docx]

**Additional file 3:**

**Table S3 Capture probes of the EMT markers used in RNA-ISH [29]**

| **Epithelial markers:** | | **Mesenchymal markers:** | |
| --- | --- | --- | --- |
| **Gene** | **Probe Sequences (5’-3’)** | **Gene** | **Probe Sequences (5’-3’)** |
| EpCAM | TGGTGCTCGTTGATGAGTCA  AGCCAGCTTTGAGCAAATGA  AAAGCCCATCATTGTTCTGG  CTCTCATCGCAGTCAGGATC  TCCTTGTCTGTTCTTCTGAC  CTCAGAGCAGGTTATTTCAG | Vimentin | GAGCGAGAGTGGCAGAGGAC  CTTTGTCGTTGGTTAGCTGG  CATATTGCTGACGTACGTCA  GAGCGCCCCTAAGTTTTTAA  AAGATTGCAGGGTGTTTTCG  GGCCAATAGTGTCTTGGTAG |
| CK8 | CGTACCTTGTCTATGAAGGA  ACTTGGTCTCCAGCATCTTG  CCTAAGGTTGTTGATGTAGC  CTGAGGAAGTTGATCTCGTC  CAGATGTGTCCGAGATCTGG  TGACCTCAGCAATGATGCTG | Twist | ACAATGACATCTAGGTCTCC  CTGGTAGAGGAAGTCGATGT  CAACTGTTCAGACTTCTATC  CCTCTTGAGAATGCATGCAT  TTTCAGTGGCTGATTGGCAC  TTACCATGGGTCCTCAATAA |
| CK18 | AGAAAGGACAGGACTCAGGC  GAGTGGTGAAGCTCATGCTG  TCAGGTCCTCGATGATCTTG  CAATCTGCAGAACGATGCGG  AAGTCATCAGCAGCAAGACG  CTGCAGTCGTGTGATATTGG |  |  |
| CK19 | CTGTAGGAAGTCATGGCGAG  AAGTCATCTGCAGCCAGACG  CTGTTCCGTCTCAAACTTGG  TTCTTCTTCAGGTAGGCCAG  CTCAGCGTACTGATTTCCTC  GTGAACCAGGCTTCAGCATC |  |  |
