# Supplementary material for: Metabolic classification of circulating tumor cells as a biomarker for metastasis and prognosis in breast cancer
Source: J Transl Med. 2020 Feb 6;18:59. doi: 10.1186/s12967-020-02237-8 (PMC7003411; doi:10.1186/s12967-020-02237-8)
Supplement: Supplementary file 4 — Additional file 4: Table S4. Parameters of the fluorescent channels in practical CTCs analysis [file 12967_2020_2237_MOESM4_ESM.docx]

**Additional file 4:**

**Table S4 Parameters of the fluorescent channels in practical CTCs analysis**

| **Channel** | **Fluorescence** | **Targets** | **Indication** |
| --- | --- | --- | --- |
| C_1_ | DAPI | Cell nucleus | Nuclear morphology |
| C_2_ | Alexa Fluor 740 | CD45 | Leukocyte marker |
| C_3_ | Cy3 | EpCAM/CKs | Epithelial (E) markers |
| C_4_ | Alexa Fluor 488 | Vimentin/Twist | Mesenchymal (M) markers |
| C_5_ | Alexa Fluor 647 | PGK1/G6PD | Metabolic (GM) markers |
