# Supplementary material for: Metabolic classification of circulating tumor cells as a biomarker for metastasis and prognosis in breast cancer
Source: J Transl Med. 2020 Feb 6;18:59. doi: 10.1186/s12967-020-02237-8 (PMC7003411; doi:10.1186/s12967-020-02237-8)
Supplement: Supplementary file 5 — Additional file 5: Figure S1. The mRNA expressions of PGK1 and G6PD in TCGA breast invasive cancer cohort. [file 12967_2020_2237_MOESM5_ESM.docx]

**Additional file 5:**

**Figure S1**

**
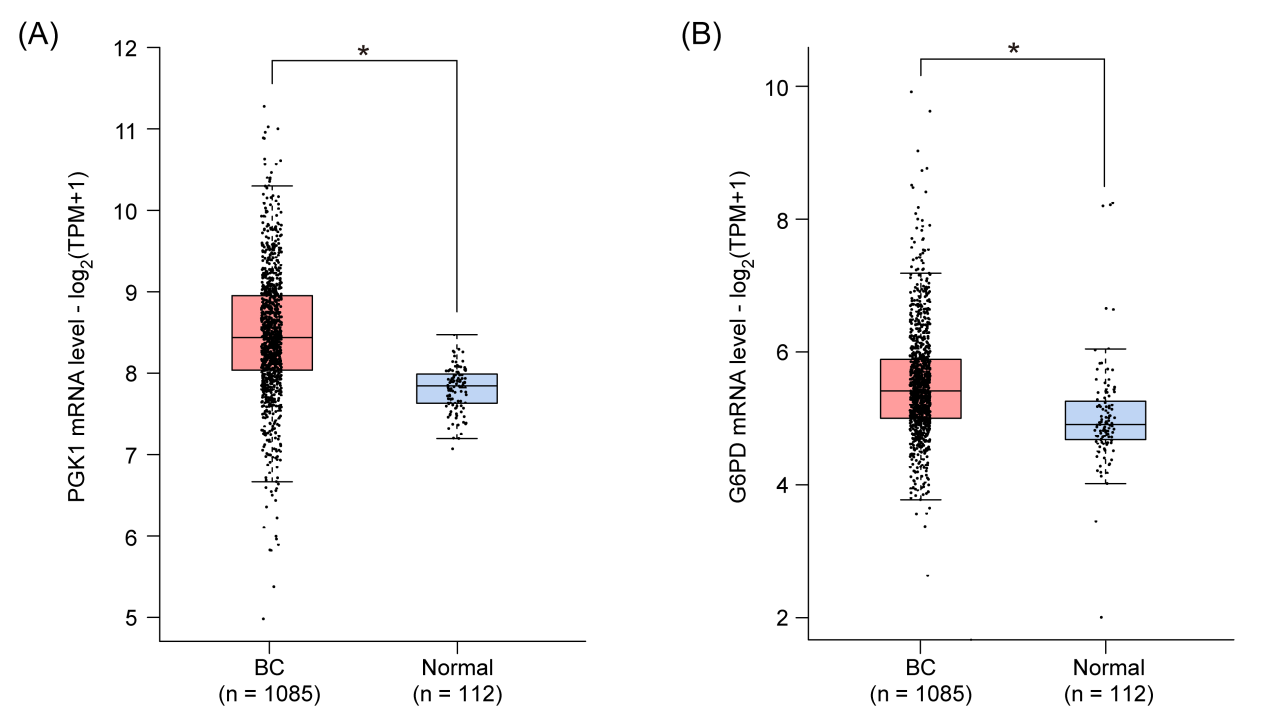
**

**Figure S1.** The mRNA expressions of PGK1 and G6PD in TCGA breast invasive cancer cohort. (A) and (B), Comparison of PGK1 (A) and G6PD (B) expression between the 1085 BC tissues and 112 normal mammary tissues. Gene expression data of the RNA-seq datasets were transformed to log_2_ (transcript count per million [TPM]+1). ^*^*P* < 0.05.
