# Supplementary material for: Metabolic classification of circulating tumor cells as a biomarker for metastasis and prognosis in breast cancer
Source: J Transl Med. 2020 Feb 6;18:59. doi: 10.1186/s12967-020-02237-8 (PMC7003411; doi:10.1186/s12967-020-02237-8)
Supplement: Supplementary file 6 — Additional file 6: Table S5. Correlation between CTCs EMT subtypes and clinical data of BC patients. [file 12967_2020_2237_MOESM6_ESM.docx]

**Additional file 6:**

**Table S5 Correlation between CTCs EMT subtypes and clinical data of BC patients**

| **Clinical characteristics** | **E-CTCs** | | **H-CTCs** | | **M-CTCs** | |
| --- | --- | --- | --- | --- | --- | --- |
| **Subgroup (n)** | **P/N^a^** | ***P*^b^** | **P/N** | ***P*** | **P/N** | ***P*** |
| Age (years) |  | 0.485 |  | 0.632 |  | 0.762 |
| ≤ 50 (n=31) | 7/24 |  | 15/16 |  | 12/19 |  |
| > 50 (n=33) | 10/23 |  | 14/19 |  | 14/19 |  |
| Histology |  | 0.846 |  | 0.220 |  | 0.850 |
| Ductal (n=52) | 13/39 |  | 26/26 |  | 22/30 |  |
| Lobular (n=6) | 2/4 |  | 2/4 |  | 2/4 |  |
| Other (n=6) | 2/4 |  | 1/5 |  | 2/4 |  |
| Tumor size |  | 0.974 |  | 0.578 |  | 0.085 |
| ≤ 5cm (n=51) | 13/38 |  | 24/27 |  | 18/33 |  |
| > 5cm (n=13) | 4/9 |  | 5/8 |  | 8/5 |  |
| Grading |  | 0.461 |  | 0.149 |  | 0.257 |
| I-II (n=35) | 8/27 |  | 13/22 |  | 12/23 |  |
| III (n=29) | 9/20 |  | 16/13 |  | 14/15 |  |
| Lymph node invasion |  | 0.380 |  | 0.107 |  | 0.098 |
| No (n=14) | 5/9 |  | 9/5 |  | 3/11 |  |
| Yes (n=50) | 12/38 |  | 20/30 |  | 23/27 |  |
| Distant metastasis |  | 0.163 |  | 0.007^*^ |  | 0.008^*^ |
| No (n=46) | 10/36 |  | 16/30 |  | 14/32 |  |
| Yes (n=18) | 7/11 |  | 13/5 |  | 12/6 |  |
| Clinical Stage |  | 0.272 |  | 0.533 |  | 0.184 |
| I-II (n=26) | 5/21 |  | 13/13 |  | 8/18 |  |
| III-IV (n=38) | 12/26 |  | 16/22 |  | 18/20 |  |
| ER expression |  | 0.199 |  | 0.033^*^ |  | 0.973 |
| - (n=22) | 8/14 |  | 14/8 |  | 9/13 |  |
| + (n=42) | 9/33 |  | 15/27 |  | 17/25 |  |
| PR expression |  | 0.171 |  | 0.169 |  | 0.935 |
| - (n=25) | 9/16 |  | 14/11 |  | 10/15 |  |
| + (n=39) | 8/31 |  | 15/24 |  | 16/23 |  |
| HER2 expression |  | 0.253 |  | 0.885 |  | 0.769 |
| - (n=16) | 6/10 |  | 7/9 |  | 6/10 |  |
| + (n=48) | 11/37 |  | 22/26 |  | 20/28 |  |
| HER2 amplification |  | 0.710 |  | 0.390 |  | 0.660 |
| - (n=39) | 11/28 |  | 16/23 |  | 15/24 |  |
| + (n=25) | 6/19 |  | 13/12 |  | 11/14 |  |

a, P: positive; N: negative. The positive criterion of E-CTCs and H-CTCs is ≥ 2/5 mL, and the positive criterion of M-CTCs is ≥ 1/5 mL. b, ^*^*P* < 0.05.
