# Supplementary material for: Metabolic classification of circulating tumor cells as a biomarker for metastasis and prognosis in breast cancer
Source: J Transl Med. 2020 Feb 6;18:59. doi: 10.1186/s12967-020-02237-8 (PMC7003411; doi:10.1186/s12967-020-02237-8)
Supplement: Supplementary file 7 — Additional file 7: Table S6. Correlation between CTCs parameters and disease progression of BC patients. [file 12967_2020_2237_MOESM7_ESM.docx]

**Additional file 7:**

**Table S6 Correlation between CTCs parameters and disease progression of BC patients**

| **CTCs parameters^a^** | **Disease progression**  **Yes/No** | **Chi-Square**  **value** | ***P*^b^** |
| --- | --- | --- | --- |
| tCTCs |  | 4.123 | 0.042^*^ |
| + (n=35) | 11/24 |  |  |
| - (n=29) | 3/26 |  |  |
| E-CTCs |  | <0.001 | 1.000 |
| + (n=17) | 4/13 |  |  |
| - (n=47) | 10/37 |  |  |
| H-CTCs |  | 1.012 | 0.314 |
| + (n=29) | 8/21 |  |  |
| - (n=35) | 6/29 |  |  |
| M-CTCs |  | 10.698 | 0.001^**^ |
| + (n=26) | 11/15 |  |  |
| - (n=38) | 3/35 |  |  |
| GM+CTCs |  | 11.178 | 0.001^**^ |
| + (n=20) | 10/10 |  |  |
| - (n=44) | 4/40 |  |  |

a, The positive criteria of these parameters were tCTCs ≥ 3/5 mL, E-CTCs ≥ 2/5 mL, H-CTCs ≥ 2/5 mL, M-CTCs is ≥ 1/5 mL and GM^+^CTCs is ≥ 2/5 mL.

b, ^*^*P* < 0.05 and ^**^ *P* < 0.01.
