# Supplementary material for: Metabolic classification of circulating tumor cells as a biomarker for metastasis and prognosis in breast cancer
Source: J Transl Med. 2020 Feb 6;18:59. doi: 10.1186/s12967-020-02237-8 (PMC7003411; doi:10.1186/s12967-020-02237-8)
Supplement: Supplementary file 8 — Additional file 8: Figure S2. Correlation between the EMT and metabolic subtypes of CTCs in BC patients. [file 12967_2020_2237_MOESM8_ESM.docx]

**Additional file 8:**

**Figure S2**

**
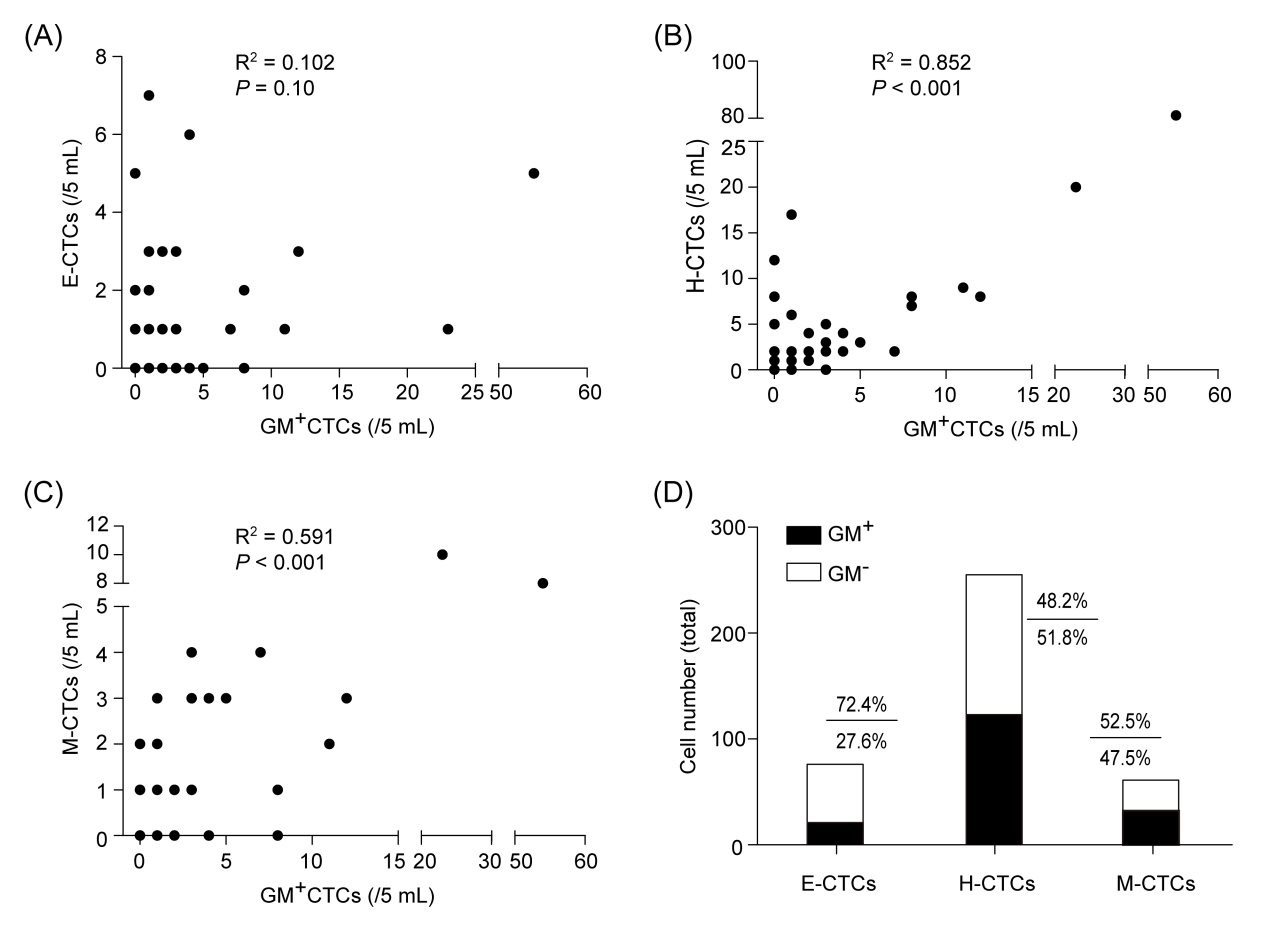
**

**Figure S2.** Correlation between the EMT and metabolic subtypes of CTCs in BC patients. (**A-C**) Correlation between the number of GM^+^CTCs with E-CTCs (**A**), H-CTCs (**B**) and M-CTCs (**C**). (**D**) Comparison of the GM^+^CTCs and GM^-^CTCs subtypes of E-CTCs, H-CTCs, and M-CTCs.
