# Supplementary material for: Metabolic classification of circulating tumor cells as a biomarker for metastasis and prognosis in breast cancer
Source: J Transl Med. 2020 Feb 6;18:59. doi: 10.1186/s12967-020-02237-8 (PMC7003411; doi:10.1186/s12967-020-02237-8)
Supplement: Supplementary file 9 — Additional file 9: Figure S3. The mRNA expressions of PGK1 and G6PD in common cancers based on TCGA RNA-seq data. [file 12967_2020_2237_MOESM9_ESM.docx]

**Additional file 9:**

**Figure S3**

**
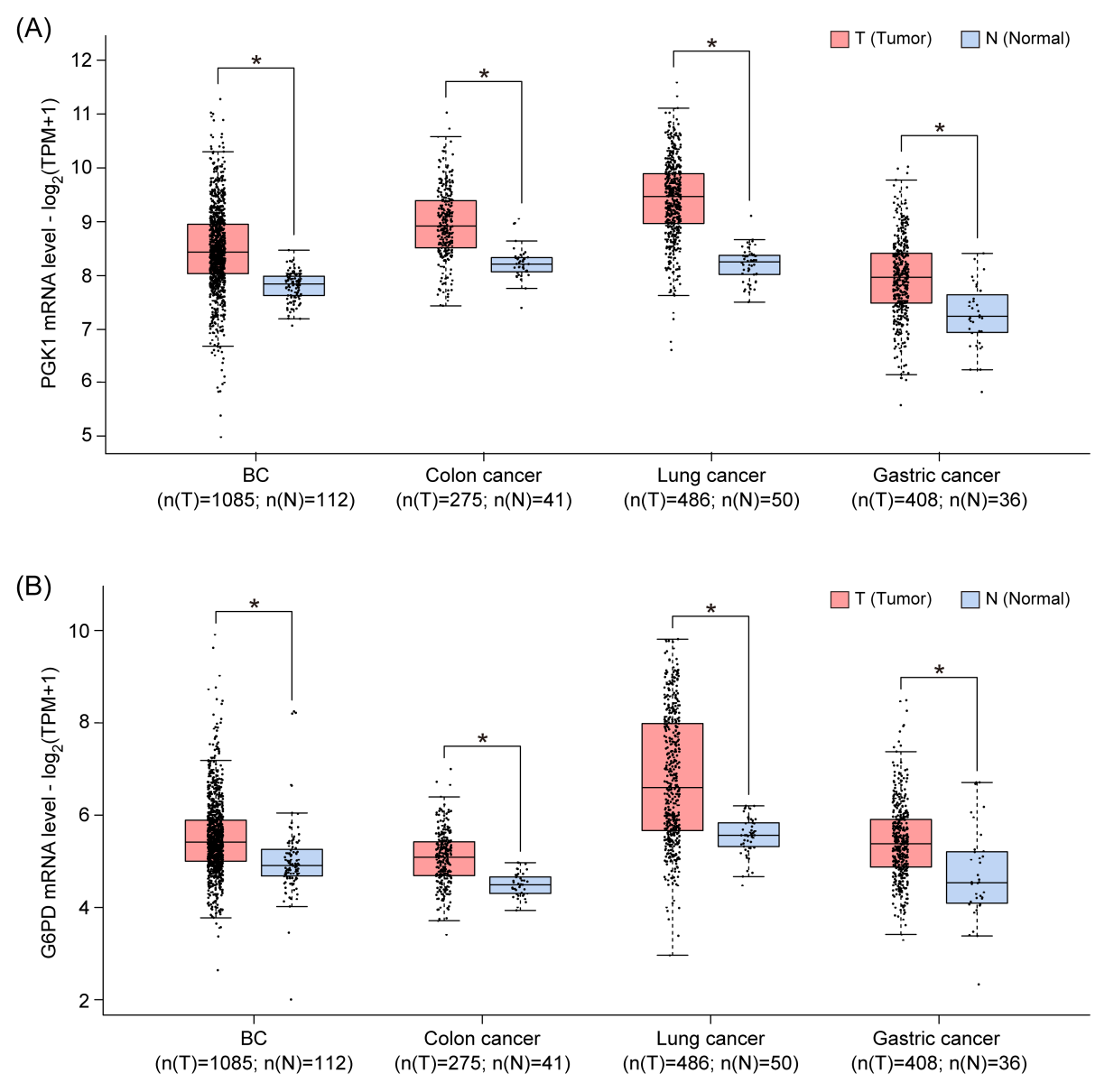
**

**Figure S3.** The mRNA expressions of PGK1 and G6PD in common cancers based on TCGA RNA-seq data. (**A**) Comparison of PGK1 expression between cancer group and normal group of breast, colon, lung and gastric cancer. (**B**) Comparison of PGK1 expression between cancer group and normal group of breast, colon, lung and gastric cancer. Gene expression data of the RNA-seq datasets were transformed to log_2_ (transcript count per million [TPM]+1). ^*^*P* < 0.05.
